# Action Potential Dynamics in Fine Axons Probed with an Axonally Targeted Optical Voltage Sensor

**DOI:** 10.1523/ENEURO.0146-17.2017

**Published:** 2017-07-25

**Authors:** Yihe Ma, Peter O. Bayguinov, Meyer B. Jackson

**Affiliations:** Department of Neuroscience, University of Wisconsin Madison, Madison, WI 53705

**Keywords:** Axonal membranes, excitability, fluorescence imaging, voltage imaging

## Abstract

The complex and malleable conduction properties of axons determine how action potentials propagate through extensive axonal arbors to reach synaptic terminals. The excitability of axonal membranes plays a major role in neural circuit function, but because most axons are too thin for conventional electrical recording, their properties remain largely unexplored. To overcome this obstacle, we used a genetically encoded hybrid voltage sensor (hVOS) harboring an axonal targeting motif. Expressing this probe in transgenic mice enabled us to monitor voltage changes optically in two populations of axons in hippocampal slices, the large axons of dentate granule cells (mossy fibers) in the stratum lucidum of the CA3 region and the much finer axons of hilar mossy cells in the inner molecular layer of the dentate gyrus. Action potentials propagated with distinct velocities in each type of axon. Repetitive firing broadened action potentials in both populations, but at an intermediate frequency the degree of broadening differed. Repetitive firing also attenuated action potential amplitudes in both mossy cell and granule cell axons. These results indicate that the features of use-dependent action potential broadening, and possible failure, observed previously in large nerve terminals also appear in much finer unmyelinated axons. Subtle differences in the frequency dependences could influence the propagation of activity through different pathways to excite different populations of neurons. The axonally targeted hVOS probe used here opens up the diverse repertoire of neuronal processes to detailed biophysical study.

## Visual Abstract

**Figure:**
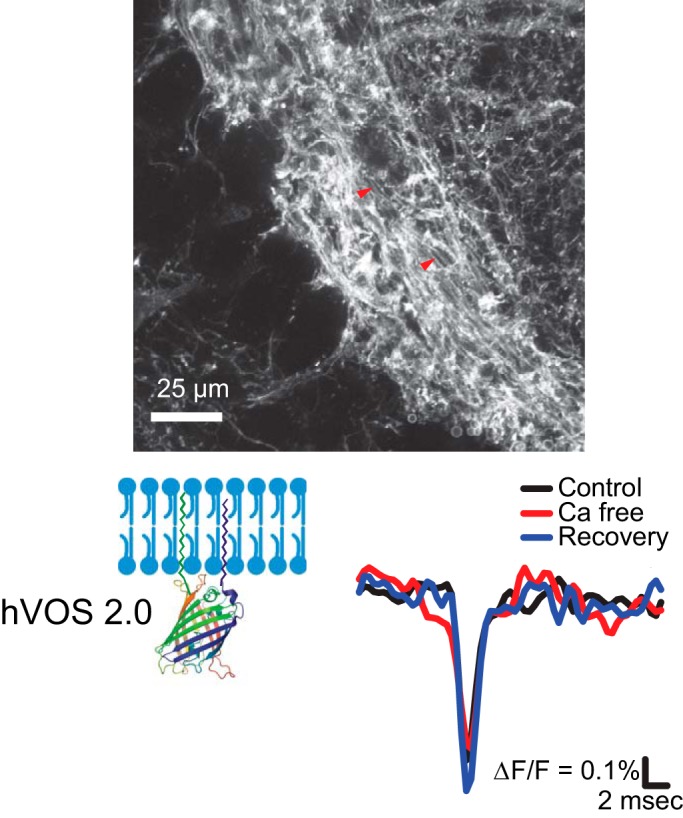

## Significance Statement

The excitability of axonal membranes plays a major role in the dynamic behavior of action potentials, determining how they invade a complex axonal arbor and how effectively they trigger synaptic release. Little is known about axonal conduction dynamics because the small size of most axons has impeded their study with electrophysiological recording techniques. We targeted a genetically encoded optical voltage sensor to axons to image their voltage changes. Focusing on two distinct populations of unmyelinated axons in hippocampal slices, imaging revealed that APs propagated with distinct velocities. Action potentials in both axons broadened and declined in amplitude during repetitive firing, but with subtle differences in frequency dependence.

## Introduction

Traditional views of neural circuits cast axons as simple transmission devices that relay temporal patterns of all-or-none action potential (AP) activity from nerve cell bodies to distant synaptic endings. However, axons can modify APs and thus contribute to neural circuit function. Activity can change axonal conduction velocity (Bullock, 1951; Soleng et al., 2004; Bucher and Goaillard, 2011; Kim et al., 2012), whereas repetitive firing (Gainer et al., 1986; Jackson et al., 1991; Geiger and Jonas, 2000; Muschol et al., 2003; Hoppa et al., 2014) and glutamate receptor activation (Sasaki et al., 2011) broaden APs to facilitate Ca^2+^ entry and synaptic release. Axonal swellings and branch points can delay or obstruct conduction, and the probability of failure depends on factors such as prior activity and receptor activation (Dudek and Blankenship, 1976; Segev, 1990; Jackson and Zhang, 1995; Obaid and Salzberg, 1996; Muschol et al., 2003; Zhang et al., 2007). These dynamic aspects of axonal excitability greatly expand the computational capacity and plasticity of the nervous system.

Axonal excitability is generally difficult to study because the vast majority of axons are too small for conventional electrophysiological recordings. Patch-clamp recording is possible in unusually large nerve terminals, such as the posterior pituitary (Jackson et al., 1991), chick ciliary ganglion (Stanley and Goping, 1991), calyx of Held (Forsythe, 1994), and mossy fibers (Geiger and Jonas, 2000), as well as lesion-induced axon blebs (Shu et al., 2006; Bean, 2007; Christie et al., 2011). Cell-attached patch-clamp recording can be performed in unmyelinated axons, but this is technically difficult (Sasaki et al., 2012). Thus, although special conditions and procedures have enabled some significant advances, there is no general, broadly applicable approach to the study of axonal conduction dynamics.

Voltage-sensitive dyes can provide insight into the excitability of axons (Ross et al., 1977; Stuart and Palmer, 2006; Canepari et al., 2015; Popovic et al., 2015). A genetically encoded voltage indicator (GEVI) has also been used to study AP waveforms in presynaptic terminals (Hoppa et al., 2014). However, synthetic dyes and GEVIs generally distribute through a cell uniformly, making it difficult to use them to study the excitability of selected subcellular compartments. Here we investigated axonal dynamics with a fluorescence resonance energy transfer (FRET)-based GEVI that preferentially targets axons. This hybrid voltage sensor (hVOS) has a rapid response and tracks APs with high temporal fidelity (Chanda et al., 2005; Wang et al., 2010; Ghitani et al., 2015). In hippocampal slices from transgenic mice expressing the axonal hVOS variant hVOS 2.0, from a pan-neuronal thy-1 promoter (Wang et al., 2012), we found that the probe targeted large mossy fiber axons of dentate granule cells and much finer hilar mossy cell axons. hVOS imaging revealed that these axons conduct APs with distinct velocities. We confirmed frequency-dependent broadening of APs in mossy fibers (Geiger and Jonas, 2000) and further found that APs of mossy cell axons also broaden. AP amplitudes declined during repetitive firing in both populations of axons. Broadening and attenuation increase with firing frequency, but the dependences on frequency were not identical. As an axonally targeted GEVI, hVOS 2.0 offers a unique opportunity to examine AP dynamics in distinct types of neurons targeted by genetic strategies (Huang and Zeng, 2013). More generally, subcellular targeting of GEVIs provides a powerful approach to the study of excitability in different neuronal processes.

## Materials and Methods

### Animals and slice preparation

The mice used in this study were *thy1*-hVOS transgenics described previously (Wang et al., 2012). These animals have hVOS 1.5– or hVOS 2.0–encoding sequences (Wang et al., 2010) on the *thy-1.2* expression cassette (Feng et al., 2000) integrated into a locus that drives expression in subsets of hippocampal neurons. hVOS 1.5 is cerulean fluorescent protein with a C-terminal truncated h-ras tag; hVOS 2.0 is hVOS 1.5 with an additional N-terminal GAP-43 tag (Wang et al., 2010).

Hippocampal slices were prepared by procedures approved by the Animal Care and Use Committee of the University of Wisconsin. 3- to 8-week-old animals of either sex were anesthetized by isoflurane inhalation and euthanized by decapitation. The brain was removed, immersed for 5 min in ice-cold cutting solution (in mm: 125 NaCl, 4 KCl, 1.25 NaH_2_PO_4_, 26 NaHCO_3_, 6 MgSO_4_, and 1 CaCl_2_ bubbled with 5% CO_2_-95% O_2_), and mounted in a cutting chamber. Horizontal slices were cut at 350–400 µm and allowed to recover in artificial CSF (aCSF; in mm, 125 NaCl, 4 KCl, 1.25 NaH_2_PO_4_, 26 NaHCO_3_, 1.3 MgSO_4_, and 2.5 CaCl_2_) containing 2–4 µM DPA at room temperature for 45–60 min.

### Voltage imaging

Voltage imaging was conducted with an Olympus BX51 microscope equipped with a 29-W, 435-nm LED light source (Prizmatix), a standard cyan fluorescent protein (CFP) filter set, and an Olympus XLUMPlanFl 20× objective (NA = 1.0). Images were acquired with a CCD-SMQ camera (Redshirt Imaging) at 2000 or 5000 fps at 80 × 80 or 26 × 26 resolution, respectively. The computer program Neuroplex (Redshirt Imaging) controlled the timing of illumination, stimulation, and data acquisition. HVOS images were acquired as averages of 5–10 trials at 10-s intervals. Brains slices were perfused with 95% O_2_/5% CO_2_-bubbled aCSF containing 2–4 µM DPA. For TEA application or calcium removal, we first established baselines for 15–30 min and then perfused with aCSF containing 10 mM TEA or calcium-free aCSF for 15–30 min. Recovery occurred ∼30 min after return to control aCSF. To stimulate slices, 0.18-ms, 75- to 200-µA current pulses were applied with a model A365 stimulus isolator (World Precision Instruments) via aCSF-filled glass electrodes. Pulse trains were generated with Clampex 9.2 (Invitrogen) running on a different computer from that used for imaging, with triggering from the imaging computer initiated by Neuroplex.

### Axon morphology

To visualize axons and evaluate probe targeting, we used two-photon and stimulated emission depletion (STED) microscopy. For STED, we prepared 100-µm slices as described above. Slices were fixed overnight in 4% paraformaldehyde at 4°C, washed with PBS 4× 15 min, blocked in 10% normal goat serum with 0.2% Triton X-100 in PBS for 1 h at room temperature, incubated in chicken anti-GFP antibody (1:250 dilution; Invitrogen) in 10% normal goat serum with 0.2% Triton-X in PBS for 48 h at 4°C, washed in PBS with 10% normal goat serum for 24 h, stained with Alexa Fluor 647 goat anti–chicken IgY secondary antibody (1:100 dilution; Abcam) for 1 h at room temperature, washed 24 h, and mounted onto slides using ProLong Diamond mounting medium (Thermo Fisher Scientific). STED images were taken at the University of Wisconsin Optical Imaging Core with a Leica SP8 3X STED Super-Resolution/Confocal microscope equipped with a Leica HC PL APO 100×/1.40 objective. Two-photon images were taken with an Olympus BX61 microscope equipped with an Ultima scanning system (Bruker Corporation) illuminated by a Chameleon Ti:Sapphire laser (Coherent) using slices that were fixed overnight in 4% paraformaldehyde at 4°C. Images were analyzed and processed in ImageJ (NIH).

### Data analysis

Regions of interest were selected manually with Neuroplex software. Fluorescence signals were divided by resting light intensity and filtered at 500 or 1000 Hz with a four-pole low-pass Butterworth filter. Amplitude, half-width, and area were analyzed with Neuroplex, Clampex 9.2, Origin 9 (OriginLab), and Igor (WaveMetrics). Statistical tests were performed with Prism (GraphPad Software) and Origin 9. For maps, raw tiff stacks were spatially filtered in Neuroplex using a center-weighted 3 × 3 low-pass filter. Stacks were inverted and temporally filtered using a two-frame walking average filter. Activity maps were constructed by subtracting the mean fluorescence of the 40-ms interval before the stimulus from the maximal fluorescence within the 10-ms window after the stimulus. Spatiotemporal maps were constructed in Volumetry G6a (G.W. Hennig) by drawing rectangular regions of interest and averaging fluorescence perpendicular to the long axis of the region of interest.

## Results

### Axonal hVOS probe expression

**Figure 1.**
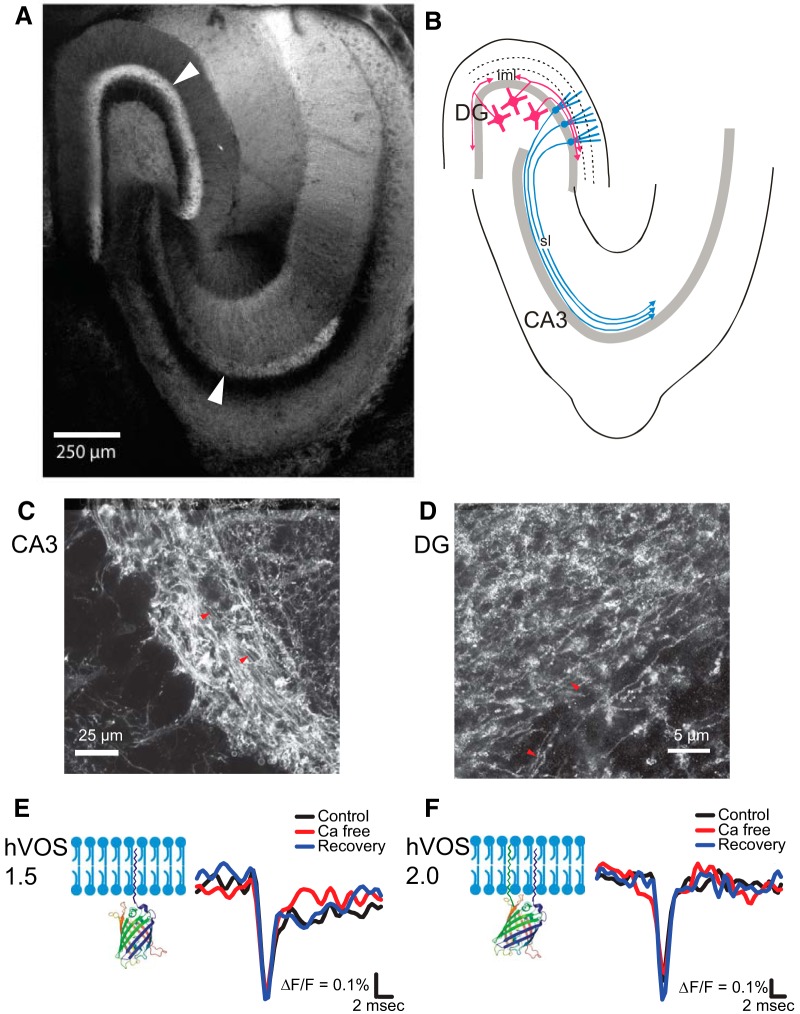
hVOS probe expression in axons. ***A***, Two-photon image of a hippocampal slice from a *thy1-*hVOS 2.0 transgenic mouse at low magnification shows the pattern of probe expression. Note the strong probe expression in the iml of the dentate gyrus (DG) and mossy fibers in the sl of the CA3 region (arrowheads). ***B***, Diagram illustrating the labeled axons in ***A*** based on known hippocampus anatomy. Axons of hilar mossy cells (magenta) are located in the iml; the sl contains axons of dentate granule cells (cyan). ***C***, At higher magnification, two-photon microscopy reveals mossy fiber axons (marked by red arrowheads) in the sl. ***D***, STED microscopy reveals probe-expressing mossy cell axons (marked by red arrowheads) in the iml. ***E***, hVOS 1.5 tethered by a C-terminal h-ras motif to the plasma membrane is illustrated on the left. In the sl, this probe registered a spike-like axonal AP followed by a synaptic response. Black, control aCSF; red, aCSF without calcium; blue, after return to control aCSF with calcium. Calcium removal reversibly blocked the later synaptic component but left the AP unchanged. ***F***, hVOS 2.0 has the same C-terminal membrane linkage as in hVOS 1.5, as well as an N-terminal link derived from GAP-43 (both termini are on the same side of the beta-barrel). hVOS 2.0 registered only an axonal AP with no late synaptic component. The AP was unaffected by calcium removal in both hVOS 1.5 and hVOS 2.0 recordings. Stimulus = 200 µA; [DPA] = 4 µM. 10-trial averages.

Standard hVOS probes have an h-ras tag at the C terminus that tethers the probe to the inner face of the plasma membrane (Chanda et al., 2005; Wang et al., 2010). hVOS 2.0 harbors an additional tag at the N terminus derived from the axonal protein GAP-43 (El-Husseini et al., 2001). As a result, neurons express this probe preferentially in axons. In *thy1-*hVOS 2.0 transgenic mice, the probe is expressed from the *thy-1.2* promoter (Feng et al., 2000), which in our line integrated into the genome at a locus that drives strong expression in the hippocampus in dentate granule cells and hilar mossy cells. In hippocampal slices from these mice, probe fluorescence was especially high in the stratum lucidum (sl) in the CA3 region, which contains mossy fiber axons of dentate granule cells. Fluorescence was also high in the inner molecular layer (iml) of the dentate gyrus, which contains axons of hilar mossy cells (Figs. 1*A*). This pattern corresponds well with the known anatomy of these axons (Fig. 1*B*). The granule cell and middle/outer molecular layers, which contain the somata and dendrites of dentate granule cells, respectively, display very little fluorescence, indicating much less probe expression in other cellular compartments. By contrast, in transgenic mice expressing hVOS 1.5, a probe identical to hVOS 2.0 but lacking the axonal targeting motif, the *thy-1.2* promoter expressed in the same types of neurons, but fluorescence was distributed more uniformly, and dendritic and somatic layers exhibited strong fluorescence (Wang et al., 2012). We confirmed axonal targeting of hVOS 2.0 with two-photon fluorescence microscopy in the sl (Fig. 1*C*) and STED microscopy in the iml (Fig. 1*D*).

Real-time optical hVOS recordings in the sl of hippocampal slices from *thy1-*hVOS 1.5 and *thy1-*hVOS 2.0 mice revealed voltage changes that were consistent with their different expression patterns. In the presence of DPA, electrical stimulation elicited fluorescence changes; depolarization drives negatively charged DPA toward the inner face of the membrane to decrease probe fluorescence through resonance energy transfer (Chanda et al., 2005). With hVOS 1.5, these signals displayed a rapid spike-like component of ∼2 ms followed immediately by a slower synaptic response. This later synaptic component was reversibly inhibited by removing calcium from the bathing medium (Fig. 1*E*). With hVOS 2.0, recordings displayed only the brief spike-like signal. The synaptic component was absent, and calcium removal had no effect (Fig. 1*F*). The absence of a postsynaptic component in recordings from hVOS 2.0 slices confirms the localization of this probe to axons and indicates that it provides a window into axonal voltage changes without interference from postsynaptic membranes.

### Action potential propagation

**Figure 2.**
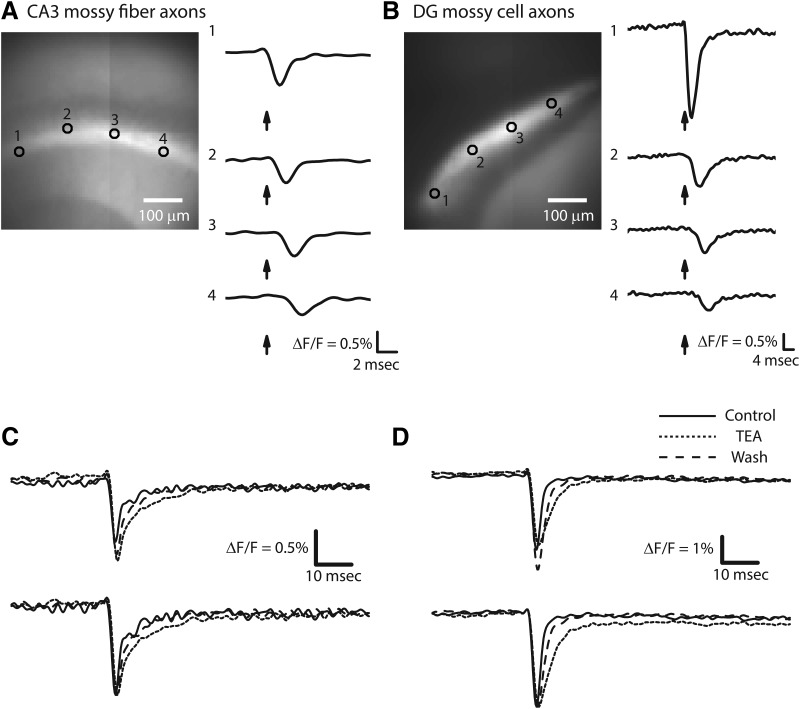
Imaging APs in a hippocampal slice from a *thy1*-hVOS 2.0 mouse. ***A***, Probe fluorescence in mossy fiber axons in the sl originating from dentate granule cells. Image shows the CA3 region (see Fig. 1*A*) with selected locations numbered in the sl. The stimulating electrode is in the sl to the left of location 1. On the right are hVOS traces (10-trial averages) from the indicated locations; arrows indicate time of stimulation. The latency increased with distance from the stimulus electrode. Stimulus = 200 µA; [DPA] = 4 µM. ***B***, Probe fluorescence in mossy cell axons in the iml. The bright iml is flanked by the darker stratum granulosum below and middle molecular layer above. The stimulating electrode is just outside the field of view from the lower left corner. Numbered locations are indicated in the iml, and traces to the right show 10-trial averages from those locations. Note the longer time scale in ***B*** compared with ***A*** due to slower propagation in mossy cell axons compared with mossy fibers. Traces in ***A*** and ***B*** were normalized. Stimulus = 200 µA; [DPA] = 4 µM. ***C***, AP broadening by 10 mM TEA in CA3 mossy fibers. ***D***, AP broadening by 10 mM TEA in mossy cell axons. Solid, control trace; dotted, in TEA; dashed, after TEA washout. TEA reversibly broadened APs by slowing repolarization. Upper traces were not normalized; lower traces were normalized to peak amplitude. Stimulus = 75 µA (***C***) and 100 µA (***D***). [DPA] = 2 µM. 10-trial averages.

Real-time imaging in a hippocampal slice from a *thy1*-hVOS 2.0 mouse showed that electrical stimulation evoked brief spike-like optical signals both in the sl, where the mossy fibers are located (Fig. 2*A*), and the iml, where the mossy cell axons are located (Fig. 2*B*). Signals were readily detected in single trials, but averaging 5–10 trials reduced the noise and produced traces with clearer dynamics. These optical signals propagated away from the site of stimulation along axonal tracks in both the sl and iml (Figs. 2*A*, *B*). Previous studies showed that hVOS signals in hippocampal slices could be blocked by the Na^+^ channel blocker tetrodotoxin (Wang et al., 2012). Here, we added the K^+^ channel blocker tetraethylammonium (TEA) to broaden these optical APs in both the sl (Fig. 2*C*) and iml (Fig. 2*D*); the broadening reversed on TEA removal (dashed traces in Figs. 2*C*, *D*). This increase in spike duration reflected a slowing of the falling phase and indicated that K^+^-channels contribute to AP repolarization. K^+^ channel blockade has a similar effect on electrically recorded APs in the large nerve terminals of the squid giant synapse and rat posterior pituitary (Augustine, 1990; Jackson et al., 1991).

**Figure 3.**
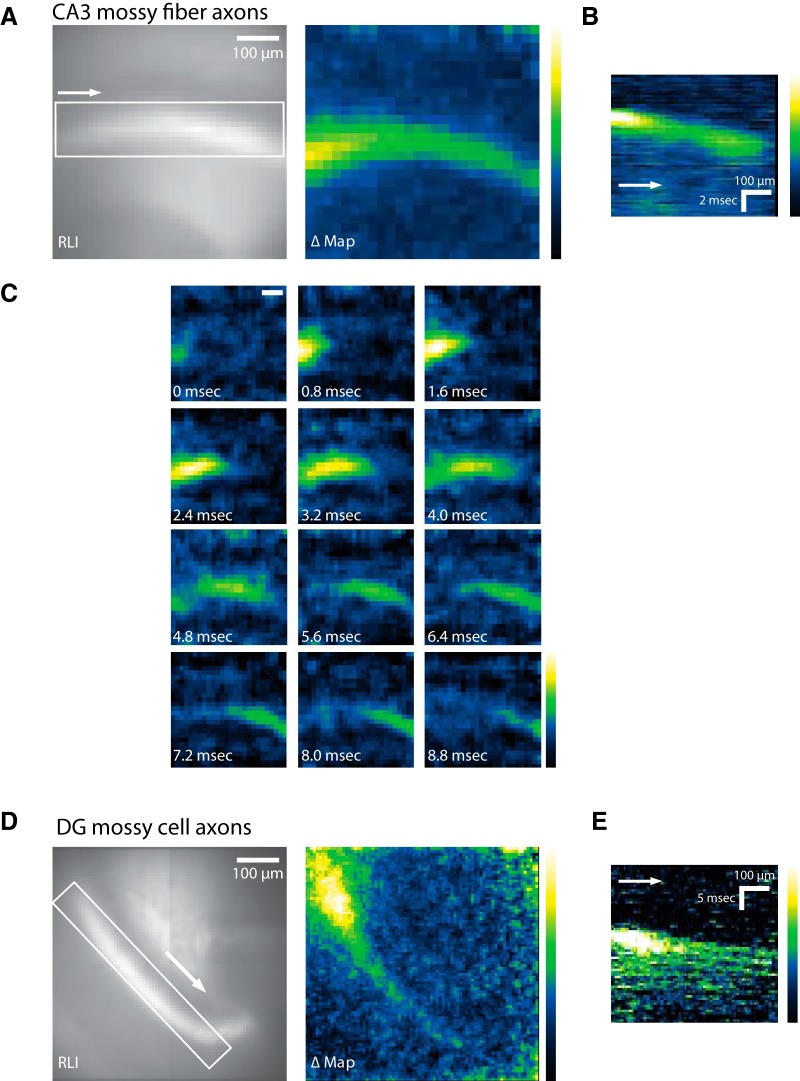
Spatiotemporal dynamics of hVOS signals. ***A***, In the CA3 region of a hippocampal slice, images of probe fluorescence [resting light intensity (RLI), left] and maximal stimulus-evoked fluorescence change (ΔMap, right) illustrate the distribution of probe and voltage response, respectively. The response map was created by subtracting the mean baseline fluorescence intensity from the maximal response to generate ΔF/F. Responses were inverted to make ΔF/F positive and aid in visualization. ***B***, Spatiotemporal map showing spread of activity in the region of interest (white rectangle) in the RLI image in ***A*** (left). Fluorescence (averaged along the *y*-axis for each *x* value) was plotted versus time to show the spread of activity. The arrow indicates orientation with respect to the RLI image. ***C***, A sequence of response intensity snapshots at 0.8-ms intervals illustrates the spread of activity in the sl along CA3 mossy fibers (from ***A***). Scale bar = 100 µm. ***D***, RLI and ΔMap (as in ***A***) from a region in the iml in the dentate gyrus. ***E***, Spatiotemporal map (as in ***B***) of signal spread along the iml. Fluorescence was averaged along the short axis of the region of interest in ***D***. The arrow indicates orientation with respect to the RLI image in ***D***.

**Figure 4.**
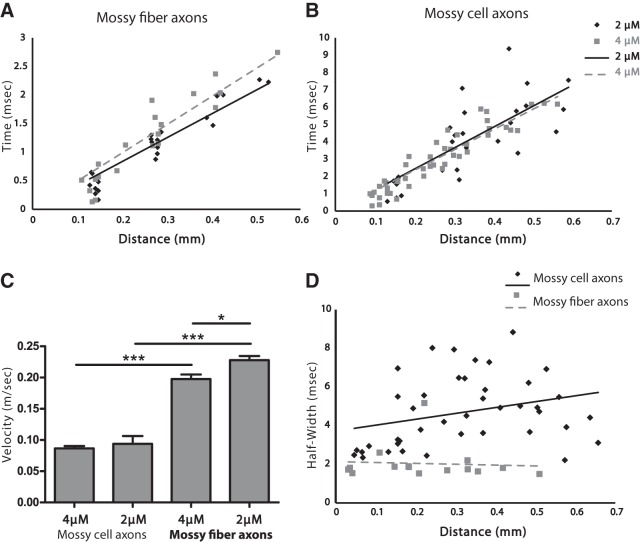
AP propagation velocity. Plots of time to half peak versus distance illustrate propagation in mossy fibers (***A***) and mossy cell axons (***B***) in 2 µM (diamonds, solid line) and 4 µM (squares, dashed line) DPA. Linearity indicates propagation with a constant velocity (*R* ^2^ = 0.902 and 0.859 in ***A***; *R* ^2^ = 0.607 and 0.870 in ***B***), and linear regression gives the velocity as the reciprocal of the slope. ***C***, Mean conduction velocities from the indicated conditions. AP conduction was faster in mossy fibers. Conduction was slightly slower in 4 µM DPA than in 2 µM; the difference was significant only in CA3 mossy fibers (*p* = 0.018; *n* = 15 and 10, respectively) but not in mossy cell axons (*p* = 0.59; *n* = 7 in both). *, *p* < 0.05; ***, *p* < 0.001. ***D***, AP half-width was correlated with propagation distance in mossy cell axons (diamonds, Spearman *r* = 0.3557, *p* = 0.0225; solid line, linear fit of the data shows a positive slope = 3.03 ms/mm), suggesting nonuniform propagation velocity. In mossy fibers (squares) there was no significant correlation between half-width and propagation distance (Spearman *r* = –0.2393, *p* = 0.3904; dashed line, linear fit of the data), suggesting more uniform conduction velocity.

Spatial maps of amplitude confirmed that optical APs followed the axonal pathways highlighted by probe fluorescence in both the sl (Fig. 3*A*) and iml (Fig. 3*D*). In the sl, a sequence of snapshots of signal amplitude illustrated the propagation of the APs along mossy fibers (Fig. 3*C*). Spatiotemporal maps of these optical APs (Figs. 3*B*, *E*) revealed a linear relation between distance and time, indicating that optical APs propagate with a constant velocity. Plotting the time to half peak amplitude versus distance also revealed a linear increase in time with distance from the site of stimulation in both mossy fibers of the sl (Fig. 4*A*) and mossy cell axons in the iml (Fig. 4*B*). The slopes of these plots provided measurements of propagation velocity (actually a lower bound because the axons are not perfectly straight). In mossy fibers, APs propagated with velocities of 0.228 ± 0.007 (*n* = 7) and 0.198 ± 0.007 (*n* = 7) mm/ms in 2 and 4 µM DPA, respectively (Fig. 4*A*). In mossy cell axons, the velocities were 0.094 ± 0.013 (*n* = 10) and 0.087 ± 0.004 (*n* = 15) mm/ms in the respective DPA concentrations (Fig. 4*B*). APs propagated more rapidly in mossy fibers than mossy cell axons, as expected for their larger diameters. Because conduction velocity scales as the square root of axon diameter (Pumphrey and Young, 1938; Huxley, 1959; Jackson, 2006), we can square the velocity ratio to estimate the diameter ratio. Doing so suggests that mossy fibers have diameters ∼5–6 times larger than mossy cell axons, although differences in axonal swellings, ion channels, and curvature could also influence these velocities. To check this result, we measured the diameters of axons in our two-photon (Fig. 1*C*) and STED (Fig. 1*D*) images and found that mossy fiber axons have diameters of 0.97 ± 0.05 µm (*n* = 7), whereas mossy cell axons have diameters of 0.19 ± 0.01 µm (*n* = 6). Their ratio of 5.1 falls within the range estimated from the square of the propagation velocities. This agreement suggests that if axon curvature influences our optical measurements of velocity, then the effect is comparable between the two populations of axons.

DPA is a charged molecule and mobile within a lipid bilayer. These properties are essential to the voltage-sensing function of hVOS imaging, but this means that DPA increases the membrane capacitance (Fernández et al., 1983; Chanda et al., 2005; DiFranco et al., 2007; Wang et al., 2010). AP conduction velocity scales with the inverse square root of capacitance (Hodgkin and Huxley, 1952; Jackson, 2006), so we expected DPA to reduce the velocity. Increasing [DPA] from 2 to 4 µM did indeed produce small reductions, but only the change for mossy fibers (Fig. 4*C*) was statistically significant (*p* = 0.017). The velocity measured in mossy fibers in 2 µM DPA of 0.228 ± 0.007 m/s was indistinguishable from the value of 0.24 m/s measured electrically in mouse hippocampal slices at room temperature (Schmidt-Hieber et al., 2008). Thus, whereas increasing the DPA concentration reduced the conduction velocity slightly, 2 µM DPA did not appear to alter the velocity from that measured electrically in the absence of DPA. This suggests that the capacitance load produced by 2 µM has minimal effects on APs. We therefore used this concentration in all the remaining experiments.

When a population of axons has heterogeneous conduction velocities, the population spike should become broader as it propagates away from the site of initiation. To assess the dispersion of velocities, we plotted the AP half-width versus distance. In the sl, the half-width showed no correlation with distance (Fig. 4*D*, from Spearman’s correlation coefficient, *p* = 0.39; *n* = 15). Thus, CA3 mossy fiber axons conduct with a relatively uniform velocity. [Our analysis of AP amplitude attenuation in mossy fibers suggested potential distortions at sites close to the site of stimulation due to direct effects of the stimulating electrode (Fig. 6*C*). However, the plot still showed no significant correlation when the most proximal points were removed.] In contrast to mossy fibers in the sl, in mossy cell axons in the iml the spike half-width exhibited a positive correlation with distance (Fig. 4*D*, from Spearman’s correlation coefficient, *p* = 0.022; *n* = 41), indicating that mossy cell axons are more heterogeneous. This heterogeneity will erode the synchrony of firing and change the character of a volley as it propagates to postsynaptic targets at greater distances.

### Action potential broadening

**Figure 5.**
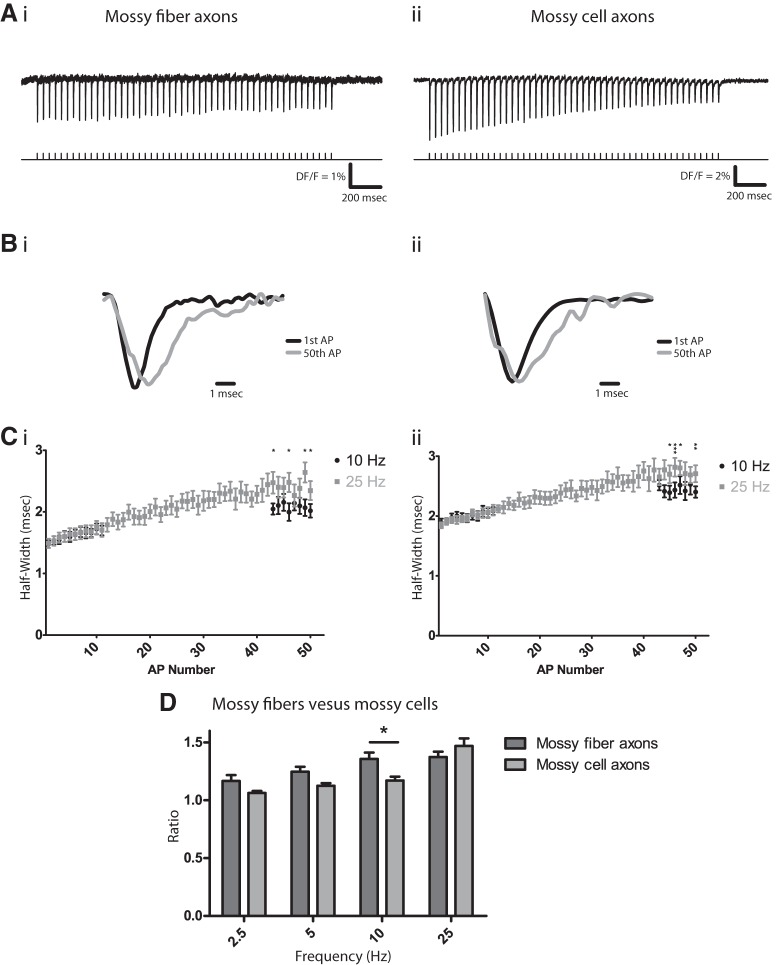
Activity-dependent AP broadening. ***A***, APs elicited by a 25-Hz train of 50 pulses in mossy fibers (i) and mossy cell axons (ii). ***B***, Superimposed normalized traces of the first (black) and 50th (gray) AP from ***A*** show that the final AP is broader, with a slower decay in both mossy fibers (i) and mossy cell axons (ii). Traces are normalized to their peak amplitude. ***C***, Half-width versus spike number at 10 Hz (black) and 25 Hz (gray) in mossy fibers (i) and mossy cell axons (ii; note that the points from the two frequencies overlie one another for the first 10 spikes). *n* = 13 and 11 for mossy fibers; *n* = 10 and 10 for mossy cell axons, where *n* is number of slices. ***D***, Frequency-dependent AP broadening differed between mossy fibers (dark gray) and mossy cell axons (light gray). AP broadening was assessed by dividing the mean half-width of the last two APs in a train of 50 by the mean half-width of the first two APs. Two-way ANOVA indicated that both frequency (*p* = 0.027) and region (sl and iml; *p* < 0.0001) contribute to the variation in broadening ratio. The interaction between frequency and region was also significant (*p* = 0.016). *p* = 0.32, 0.084, 0.0084, and 0.12 (in order of increasing frequency) for the *post hoc* tests, compared with *p* = 0.0125 with the Bonferroni correction. *n* = 11, 16, 13, 20 slices for mossy fibers; *n* = 9, 10, 12, 13 slices for mossy cell axons (increasing frequency). Stimulus = 100 µA; [DPA] = 2 µM. *, *p* < 0.05; **, *p* < 0.01; ***, *p* < 0.001.

Fig. 4*D* indicates that APs are more than twice as broad in mossy cell axons than mossy fiber axons. This difference in duration is likely to carry over to AP efficacy in eliciting Ca^2+^ entry and transmitter release at synapses (Augustine, 1990). Repetitive firing can increase release by broadening APs, as has been shown in the large nerve terminals of the posterior pituitary (Gainer et al., 1986; Jackson et al., 1991) and in mossy fibers (Geiger and Jonas, 2000). Here we found that APs recorded with hVOS 2.0 also broadened during repetitive activity. The sl was stimulated repetitively at 25 Hz (Fig. 5*Ai*), and superposition of the first and 50th spikes (normalized) revealed a clear broadening (Fig. 5*Bi*). The 50th spike repolarized more slowly, consistent with the established role of K^+^ channels (Aldrich et al., 1979; Jackson et al., 1991; Geiger and Jonas, 2000; and the present results on AP broadening by TEA; Figs. 2*C*, *D*). We assessed the frequency dependence of AP broadening using trains of 50 spikes at four different frequencies ranging from 2.5 to 25 Hz. Bursts of this duration and frequency roughly correspond with observed spike trains recorded in freely moving rodents (Klyachko and Stevens, 2006). Fig. 5*Ci* plots the AP half-width versus spike number during trains of 10 and 25 Hz. At both frequencies APs broadened progressively over the course of the trains. By the end of the train, there was clearly more broadening at 25 Hz than at 10 Hz (Fig. 5*Ci*). [Note that with 10 Hz or less the time needed to record a 50 spike train (≥5 s) exceeded the capability of our data acquisition system for continuous recording, so we paused after recording the first 11 APs and resumed recording for the final 8 APs of the train.] Comparison of the half-widths of the first two and last two APs indicated significant broadening at all four frequencies tested and more broadening at higher frequency (Fig. 5*D*, dark gray bars).

As in mossy fiber axons, high-frequency stimulation also broadened APs in mossy cell axons. Half-widths were greater at the end of a train than at the beginning (Figs. 5*Aii*, *Bii*), and the increase developed progressively over the course of the train, as illustrated for 10 and 25 Hz (Fig. 5*Cii*). This broadening was seen over the entire range of frequencies studied (2.5–25 Hz; Fig. 5*D*), with increased broadening at higher frequency. However, in mossy cell axons, AP broadening at low frequencies was relatively weak compared with that in mossy fiber axons (Fig. 5*D*). We compared AP broadening between these two populations of axons by performing two-way ANOVA on the ratio of the half-widths of the last two APs to the first two APs. Region and frequency each significantly influenced this ratio (*p* = 0.027, *p* < 0.0001, respectively), and the interaction between region and frequency was also significant (*p* = 0.016). A *post hoc* test for the two axon types confirmed this difference at 10 Hz (*p* = 0.0084). Thus, although both axons have dynamic properties that can support use-dependent facilitation, subtle differences enable mossy fiber APs to broaden more at lower frequencies.

### Action potential failure

**Figure 6.**
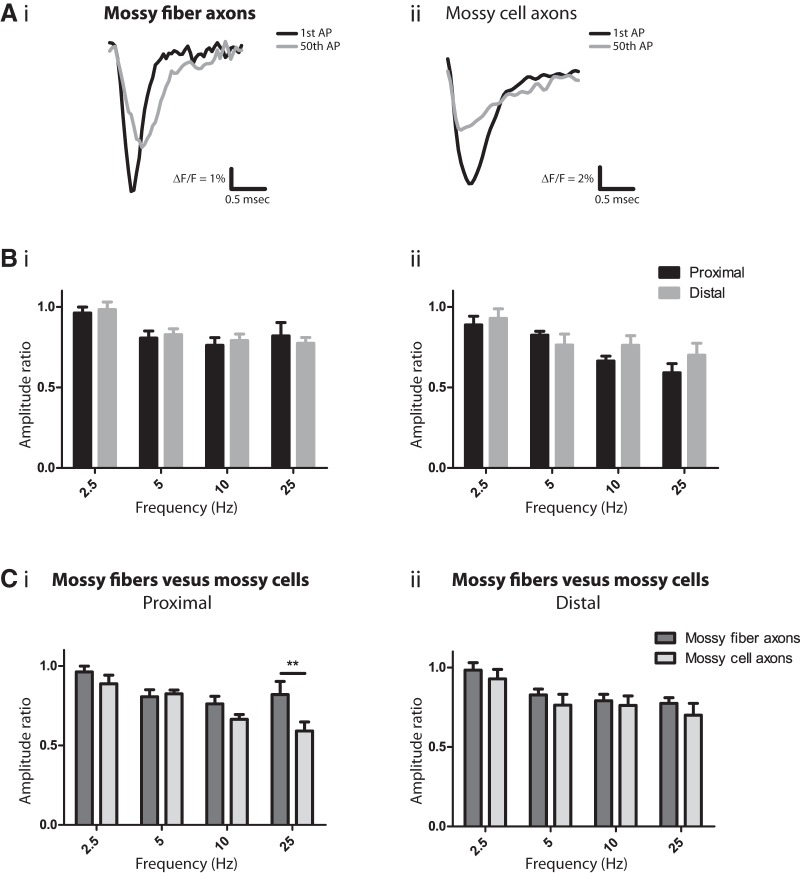
Activity-dependent AP attenuation. ***A***, Superimposed recordings (not normalized) of the first (black) and last (gray) AP from a 25-Hz train of 50 spikes from mossy fibers (i) and mossy cell axons (ii) showed that the final APs were smaller. ***B***, AP amplitudes decline in mossy fibers (i) and mossy cell axons (ii) assessed by amplitude ratio near the stimulation site (proximal; black) and ∼300 µm away (distal; gray). Ratios were calculated as the average amplitude of the last two APs divided by the average amplitude of the first two APs. Amplitude decreased during repetitive firing, and this decrease was greater at higher frequencies (two-way ANOVA, *p* = 0.0062 for mossy fibers and 0.0008 for mossy cell axons). The changes at proximal and distal sites were statistically indistinguishable (*p* = 0.80 in mossy fibers; 0.20 in mossy cells). ***C***, Comparisons of frequency-dependent amplitude decrease between mossy fibers (dark gray) and mossy cell axons (light gray) at proximal (i) and distal sites (ii). Two-way ANOVA indicated that near the stimulation site, both axon type and frequency contributed to use-dependent decreases (*p* = 0.0069 and *p* < 0.0001, respectively). *p* = 0.26, 0.76, 0.18, and 0.0020 (in order of increasing frequency) for the *post hoc* tests, compared with *p* = 0.0125 and 0.0025 with the Bonferroni correction (**, *p* < 0.01). At distal sites, however, the decreases were similar in the two axon types (*p* = 0.19). *n* = 9, 11, 7, 7 for CA3 mossy fibers and *n* = 11, 12, 9, 11 for mossy cell axons (increasing frequency). Stimulus = 100 µA; [DPA] = 2 µM.

The amplitudes of optical APs became smaller with repetitive firing. Superimposing traces of the first and 50th spikes without normalization illustrated that the amplitudes declined in both mossy fibers (Fig. 6*Ai*) and mossy cell axons (Fig. 6*Aii*). Because these population responses reflect APs in many individual axons, the reduction could reflect AP failures in significant numbers of axons after repetitive activity. However, because reductions in AP amplitude in individual axons could also contribute (Hoppa et al., 2014), we cannot determine whether the reductions in amplitude are caused by AP failure in individual axons. Area generally declined in parallel with amplitude (data not shown), indicating that the reductions do not reflect a loss of synchrony (as occurs when APs propagate with a nonuniform conduction velocity in mossy cell axons, Fig. 4*D*). Furthermore, a loss of synchrony should broaden the wave form symmetrically, but the broadening we observed was asymmetric (Figs. 5*B* and 6*Ai*, *Aii* ). AP broadening will increase the area of the wave form and thus lessen the apparent decrease in amplitude. However, broadening will have little effect on amplitude. Both amplitude and area decreased in parallel, but with occasional small differences. In these cases, the reductions in area were always less, presumably due to a small counteracting effect of AP broadening. Overall, amplitude provided a better measure of use-dependent degradation of APs, so we focused our attention on amplitude rather than area.

AP amplitude attenuation could reflect either an increase in threshold at the site of initiation or a block of conduction. To distinguish between these two possibilities, we analyzed responses locally, near the site of stimulation (<50 µm) and distally (∼300 µm away). At both sites, higher frequencies produced greater declines in amplitude in both mossy fiber axons (Fig. 6*Bi*) and mossy cell axons (Fig. 6*Bii*). The parallel declines proximally and distally indicate that repetitive activity raises the threshold for AP initiation, and that once initiated, these axons conduct APs reliably over the distances within view, as well as over the range of frequencies tested. In comparing the two populations of axons, two-way ANOVA indicated that the AP amplitude declines differed between these two types of axons at proximal sites (Fig. 6*Ci*, *p* = 0.0069), but not at distal sites (Fig. 6*Cii*, *p* = 0.19). We saw a significant difference in the amplitude ratio only at 25 Hz and only at proximal sites (*p* = 0.0020). It is surprising that the difference at proximal sites does not carry over to distal sites. The distal amplitude ratio was only slightly smaller in mossy cell axons, and the difference was not statistically significant. One possible explanation is that some of the proximal axons are directly depolarized by the stimulating electrode. Summation of this direct voltage change with the AP could dampen an excitability-related decrease in amplitude. In fact, based on the well-established dependence of stimulus-induced voltage changes on neuron size (Tehovnik et al., 2006), we would expect the direct effect to be greater in mossy fibers because of their larger diameter. This is a small effect, but it makes the distal measurements more reliable than the proximal measurements and further indicates that with regard to use-dependent declines in amplitude, the two populations of axons are similar. These data suggest that in both mossy fiber and mossy cell axons the threshold for spike initiation increases during repetitive firing, that conduction is reliable, and that the frequency dependences of amplitude declines in the two axons are probably similar.

## Discussion

This study has introduced a hybrid-GEVI that targets axons and enables imaging techniques to be used to investigate axonal dynamics. We confirmed previous work in large diameter mossy fibers on conduction velocity (Schmidt-Hieber et al., 2008) and AP broadening (Geiger and Jonas, 2000). Turning to the axons of hilar mossy cells, which are much too fine for direct electrical recording, we broke new ground in characterizing the dynamics of AP conduction, broadening, and attenuation. Both types of axons can facilitate and fatigue to a degree that depends on frequency. APs broadened somewhat more in mossy fiber axons than in mossy cell axons. Because AP broadening reflects slower repolarization (Fig. 5), which can result from K^+^ channel inactivation (Aldrich et al., 1979; Jackson et al., 1991), the difference could be explained by different K^+^ channels. At frequencies up to 10 Hz, the K^+^ channels of mossy fibers may recover less from inactivation between spikes. The similar AP broadening at 25 Hz would then suggest that at that frequency, the gap in K^+^ channel inactivation had closed. K^+^ channels vary markedly in their inactivation properties; some inactivate only partially and some not at all. Axons likely have both inactivating and non-inactivating components of K^+^ current. The AP repolarization seen at high frequency (25 Hz) could then reflect more noninactivating K^+^ current.

Mossy fiber axons express a number of voltage-gated K^+^ channel subunits, including K_v_ 1.1, 1.4, 3.2, 3.3, 7.2, and 7.3, along with K_Ca_1.1 α subunits (Trimmer and Rhodes, 2004; Vacher et al., 2008; Trimmer, 2015). Less is known about mossy cell axons. K_v_3.1β subunits have been detected in the iml; K_v_1 and K_Ca_ channels were much less abundant (Vacher et al., 2008; Trimmer, 2015). In mossy fiber boutons, high voltage-activated, slowly inactivating K_v_3 channels dominate in AP repolarization (Alle et al., 2011), but rapidly inactivating K_v_1 channels may also contribute (Geiger and Jonas, 2000). Modulation of K^+^ channels through a retrograde action of arachidonic acid may also influence the AP wave form in mossy fibers (Carta et al., 2014).

AP amplitudes decline during repetitive stimulation. The similar attenuation at both proximal and distal sites indicated that conduction is relatively reliable but that initiation is more sensitive. Just as K^+^ channel inactivation can broaden APs, K^+^ channel enhancement can make them fail or reduce their amplitude. NO/cGMP-mediated K^+^ channel modulation contributes to propagation failure in pituitary axons (Obaid and Salzberg, 1996; Zhang et al., 2007), and modeling has shown that axonal varicosities act as obstacles to AP propagation (Segev, 1990; Jackson and Zhang, 1995). Thus, both geometry and ion channels could contribute. Mossy fibers project in the transverse plane to the CA3 region, whereas mossy cell axons project to the iml along the longitudinal axis of the dentate gyrus as well as contralaterally (Amaral et al., 2007). Given the lengths of these projections, reliable conduction will ensure that APs reach distal targets. Signaling to distal targets is also affected by the dispersion of conduction velocities (Fig. 4*D*), which degrades the synchrony of a volley and makes it less effective when postsynaptic firing depends on coincident activation by multiple inputs. This observation raises the interesting scenario that the window for temporal summation of multiple synaptic inputs can depend on propagation distance, even when conduction itself is reliable.

Voltage-sensitive dye imaging has revealed differences between the axons of specific types of cortical interneurons, and these differences are adapted to their biological functions (Casale et al., 2015). The maintenance of AP wave form is critical in nerve terminals that require high-fidelity transmission such as the calyx of Held (Ishikawa et al., 2003). On the other hand, variations in AP wave form can expand the computational capacity of neural circuits. In mossy fiber–CA3 synapses, AP broadening enhances synaptic transmission (Geiger and Jonas, 2000), potentially setting a threshold for long-term potentiation. The trains tested here cover a range of frequencies and durations observed in the hippocampus *in vivo* (Klyachko and Stevens, 2006). Different frequency ranges for AP broadening could determine which synapses of the dentate gyrus become potentiated (Wright and Jackson, 2014), whether excitatory or inhibitory neurons are more strongly activated (Mori et al., 2004; Mori et al., 2007; Jinde et al., 2013; Scharfman and Myers, 2013), whether seizure activity is amplified or contained, and how the dentate gyrus gates information flow through the hippocampus (Hsu, 2007).

Imaging with an axonally targeted probe enabled us to confirm the established properties of one well-studied population of axons, dentate granule cell mossy fibers, and characterize a much less accessible population of axons originating from hilar mossy cells. Our probe, hVOS 2.0, can monitor electrical activity in fine axons with high temporal resolution and provide a window into the excitability and dynamics of unmyelinated axons in intact circuits. Our findings suggest that AP broadening and failure may be widespread axonal properties, and future studies targeting hVOS 2.0 to specific cell types using Cre-lox technologies and other targeting techniques (Huang and Zeng, 2013) will determine how general these properties are. Furthermore, the subcellular targeting of a voltage probe has many additional potential applications for the study of other cellular compartments such as somata, dendrites, and specialized dendritic segments.

## Synthesis

Reviewing Editor: Christophe Bernard, INSERM & Institut de Neurosciences des Systèmes Decisions are customarily a result of the Reviewing Editor and the peer reviewers coming together and discussing their recommendations until a consensus is reached. When revisions are invited, a fact-based synthesis statement explaining their decision and outlining what is needed to prepare a revision will be listed below. The following reviewer(s) agreed to reveal their identity: Dejan Zecevic.

Authors should address the following points before the paper becomes suitable for publication in eNeuro:

1. Please label panels 1C and D, indicating axons (and other structures if appropriate). Also, use this figure to document the diameter of mossy fiber axons compared to mossy fiber axons

2. Given that measurements at proximal sites of the axon are suggested to be distorted by direct stimulation, the measurement at 0 mm in figure 4D should be removed before calculating the slope and concluding that the signal increases with distance

3. Since the authors suspect that the amplitude ratio at 2 Hz in figure 6Ci might be an electrode artifact, these data should be removed, unless this possibility is experimentally eliminated

4. Speculations about use-dependent plasticity of spike propagation in the discussion should be limited

5. Since axons are rather curvy, estimates of diameter ratios based on conduction velocity or the relationship between distance and time in AP propagation are imprecise. This point should be discussed

6. Figure 5ii shows 2 frequencies, not a frequency range as stated in Line 270-271.

7. State that this study does not provide direct evidence that reductions in AP amplitude are cause by AP failure in individual axons.
